# Optimized PCF architectures for THz detection of aquatic pathogens: Enhancing water quality monitoring

**DOI:** 10.1371/journal.pone.0317533

**Published:** 2025-01-27

**Authors:** Diponkar Kundu, Nasir Uddin Badhon, A. H. M. Iftekharul Ferdous, Md. Safiul Islam, Md. Galib Hasan, Khalid Sifulla Noor, Most. Momtahina Bani

**Affiliations:** Department of Electrical and Electronic Engineering, Pabna University of Science and Technology, Pabna, Bangladesh; DIT University, INDIA

## Abstract

Waterborne bacteria pose a serious hazard to human health, hence a precise detection method is required to identify them. A photonic crystal fiber sensor that takes into account the dangers of aquatic bacteria has been suggested, and its optical characteristics in the THz range have been quantitatively assessed. The PCF sensor was designed and examined as computed in Comsol Multiphysics, a program in which uses the method of “Finite Element Method” (FEM). At 3.2 THz operating frequency, the proposed sensor performs better than the others in all tested cases, with a high sensitivity of 96.78% for *Vibrio cholera*, 97.54% for *E. coli*, and 97.40% for Bacillus anthracis. It also has a very low CL of 2.095 × 10^−13^ dB/cm for *Vibrio cholera*, 4.411 × 10^−11^ dB/cm for *E. coli*, and 1.355 × 10^−11^ dB/cm for *Bacillus anthracis*. The existing architecture has the potential to produce the sensor efficiently and scalable, opening the door for commercial applications. The innovation is in the optimization of structural parameters to increase the fiber’s sensitivity to bacterial presence, thereby improving the interaction between terahertz waves and bacterial cells. It targets bacterial macromolecule absorption peaks to increase sensitivity. Localized field augmentation, which concentrates THz vibrations where bacteria interact more, may arise from optimization. By improving scattering, structural alterations can help identify bacteria by their characteristic scattering patterns. These improvements improve the sensor’s trace bacteria detection. These factors increase the sensor’s aquatic germ detection when combined. In aqueous environments, this results in a more precise and efficient detection, which could facilitate the real-time monitoring of bacterial contamination. Public health and water quality control may be significantly affected by these developments.

## 1. Introduction

There are numerous locations where microbes can be found, such as the surface and bottom of streams. Certain ones of these microorganisms may have detrimental effects on human health. Numerous epidemics have been caused by waterborne infections. Diarrhea triggered by a wide range of protozoa, viruses, and bacteria in addition to additional gastrointestinal issues. Globally, water quality is being jeopardized by the pollution of water sources by pathogens in the water and the diseases which arise as a result [1]. The three most common bacteria that contaminate water are *E. coli*, *vibrio cholera*, and *Bacillus anthracis*. Symptoms such as nausea, vomiting, diarrhea, cramps, and stomach discomfort are the result of these bacteria. In 1998, *E. coli* O157:H17 was identified in the public drinking water of Alpine, Wyoming, USA. This discovery resulted in an outbreak that affected 157 individuals, resulting in 2,300 individuals becoming ill and 7 individuals passing away in Walkerton, Ontario, Canada in 2000. The contamination of the drinking water by *E. coli* O157:H17 was the cause of the outbreak [2]. A form of electromagnetic radiation that is classified as THz radiation is that which is ranging from 0.1% to 10% of terahertz [3]. The THz region is of significant importance for scientific, military, and commercial applications due to its distinctive properties and the most recent developments in practical THz sources of energy and sensors. For the transmission of terahertz communications, PCFs are of paramount importance. Fiber optic cables that are encircled by a photonic crystal cladding are referred to as photonic crystal fibers (PCFs). PCF sensors are superior to other sensors in a variety of sensing applications due to their unique characteristics, which include an increased sensitiveness relative to other factors, reduced CL, and versatile light direction [4]. As per the article by Cerqueira, PCF enhances one that design, production, and utilisation of optic cable technology by directing light across a wide wavelength spectrum [5]. Through the removal of optical loss and the facilitation the efficient administering and controlling of light, optical fibrers have revolutionized messaging across great distances [6]. Additionally, PCF is employed in numerous applications, including the identification of microorganisms in water in biosensors for the chemical industry [7], nonlinear devices [8], temperature sensing [9], spectroscopy [10], sensing [11], Fiber lasers and distant sensing [12] etc. Using several methods such as IMD and EMD in sensing methodologies, Shakya and Singh employed PCF [13]. Recently, Safiul et al. outlined a PCF detector also inside THz region [14]. Dubious parameters are determined in the oscillations in terahertz. These days, PCFs are essential to the transmission of terahertz [15]. For solvent/synthetic detection, Detectors built from PCFs have been supported via professionals and scientists conducting the last 20 years. Waterborne bacteria can be detect by using various methods such as Colorimetric and electrochemical techniques [16], surface-enhanced Raman scattering (SERS) [17], the dual-sample on-chip LAMP [18], and laser-induced fluorescence technologies [19] etc. Despite the accuracy and sensitivity of these methods, their numerous shortcomings such as costly apparatus, intricate instrumentation, and the need for skilled operators keep them from being widely applied in resource-constrained environments, making them inaccessible for decentralized or large-scale applications. Certain techniques may also process data more slowly and have a lower throughput. In contrast to these techniques, photonic crystal fibers (PCF) provide higher sensitivity, a wider wavelength range, and more design flexibility. Additionally, they are more readily incorporated into small and lightweight devices, which makes them perfect for a variety of field and application uses. Therefore we use PCF for sensing the waterborne bacteria. Earlier, many types of sensors were proposed for various biosensor types, such as metasurface-based biosensors [20] and others that detect human sperm [21], SARS-CoV-2 [22], dentin, enamel, and cementum layer in human teeth [23], cancer cells [24], Brain Tumor Diagnosis [25] and pregnancy [26].In before, some people work on detection of waterborne bacteria such as A Numerical Study of a Biosensor Based on PCF designed by Haque et al. in 2022 [27], PCF based Proteomic Ligand Biosensor through Shrivastava et al. in 2023 [28], Detector Based on PCF Encased in Gold stated via Haque et al. in 2022 [29], refractometric Sensing of Waterborne Pathogens indicated from Photonic Crystal Fiber Sensor outlined with Rahman et al. in 2023 [30], Suthar et al. proposed Improved optical sensor for photographic crystals based on water-borne bacteria [31], In 2024 Khodaparast mentioned Significant progress in molecular and point-of-care diagnostics [32], Shrivastava et al. (2023) presented in A Surface Plasmon Resonance Biosensor Based on Holographic Crystal Fiber [28], A high-performance biosensor design recommended by Zaman et al. [33], Conceptual investigation of a one-dimensional flaw photonic crystal for use as a very effective bacteriometer in water proposed by Shalaby et al. [34], Using a photonic crystal with extremely high sensitivity, we have designed a new optical sensor for detecting bacteria in water indicated from Daher et al. [35], Detecting germs in water using a transparent and adjustable plus-shaped refractive index sensor based on graphene recommended by Patel et al. [36] etc. The detection of *E. coli*, *Bacillus anthracis*, and *Vibrio cholera* in potable water is the subject of this article. It is possible for any of these microbes to cause significant damage to our health. Stasis water, potable water, recreational water, and natural water bodies all contain these pathogens. They can result in severe illness, such as cholera and typhoid fever, as well as infections of the epidermis, respiratory tract, and gastrointestinal tract (such as Legionnaires’ disease and diarrhea). If waterborne germs cause infections such as cholera, typhoid fever, and legionnaires’ disease, they can result in mortality through severe dehydration, organ failure, or sepsis. Without treatment, these illnesses can rapidly deteriorate, particularly in individuals who are already susceptible. Having access to pure water and timely medical care is crucial for prevention. Proper water treatment and sanitation are indispensable in order to prevent these illnesses. For the purpose of detection, these PCF-based sensors are implemented. Innovative applications that enhance efficiency, productivity, and safety in numerous sectors are facilitated by a waterborne bacteria detector that employs PCF. This technology can ensure that drinking water meets rigorous regulatory standards and is safe for public consumption by enabling the instant detection of microbial contamination in municipal the water systems. To safeguard public health and ecosystems, as well as to prevent pollution events from occurring too late, environmental organizations can install these detectors in naturally occurring water bodies, including rivers, lakes, and oceans. This allows them to consistently monitor for bacterial contamination. PCF-based detectors can mitigate the risk of hospital-acquired infections in healthcare facilities such as hospitals and clinics by ensuring that the water utilized in medical procedures is devoid of hazardous microorganisms. In addition, within industrial environments, particularly in the food processing sector. The detection device we have developed is highly recommended due to its exceptional identification capabilities. Two significant guiding properties in the detection circumstance are CL and relative sensitivity. PCF’s effective area is yet another noteworthy characteristic. A greater NA is observed when the value of this parameter is lower. Similar to this, the spot size and effective material loss of PCF are significant characteristics. *Vibrio cholera*, *Bacillus anthracis*, and *E. coli* exhibit maximum relative survival (RSS) values of 97.34%, 96.78%, and 97.30%, respectively, when used with the recommended sensors. The most efficient operating frequency for our proposed sensor is determined to be within the terahertz range of 2.6 to 3.8 THz. The 2.6 to 3.8 THz range is utilized for PCF-based waterborne bacterial detection due to its high sensitivity, molecular profiling, and exceptional water absorption. This enables the efficient differentiation and identification of bacteria. In addition, this range guarantees interference-free, secure, accurate, and non-ionizing detection. It is likely that the most effective frequency range for PCF material communication is 2.6–3.8 THz, which yields effective signal transmission and reception. For the three RI, the NA values of this detector were 2.934. The previously described cells have EA values of 9.56 × 10^−01^ m^2^, 9.49 × 10^−01^ m^2^, and 9.55 × 10^−01^ m^2^.The ability to continuously monitor of the PCF detectors facilitate the rapid identification of changes in *E. coli*, *Vibrio cholera*, and *Bacillus anthracis*, thereby facilitating a timely response to any current or prospective health hazards. These sensors are excellent being used for a range of purposes, such as the monitoring of indoor workplaces and outdoor environments, as a result of their mobility and diminutive size. Developed by this method, the sensors are ideal instruments for swiftly the identification of *E. coli*, *Vibrio cholera*, and *Bacillus anthracis* in a variety of situations due to their high detection level, affordability, and ability to detect PCF. Consequently, surveillance was enhanced.

## 2. Methodology

**Fig 1:**
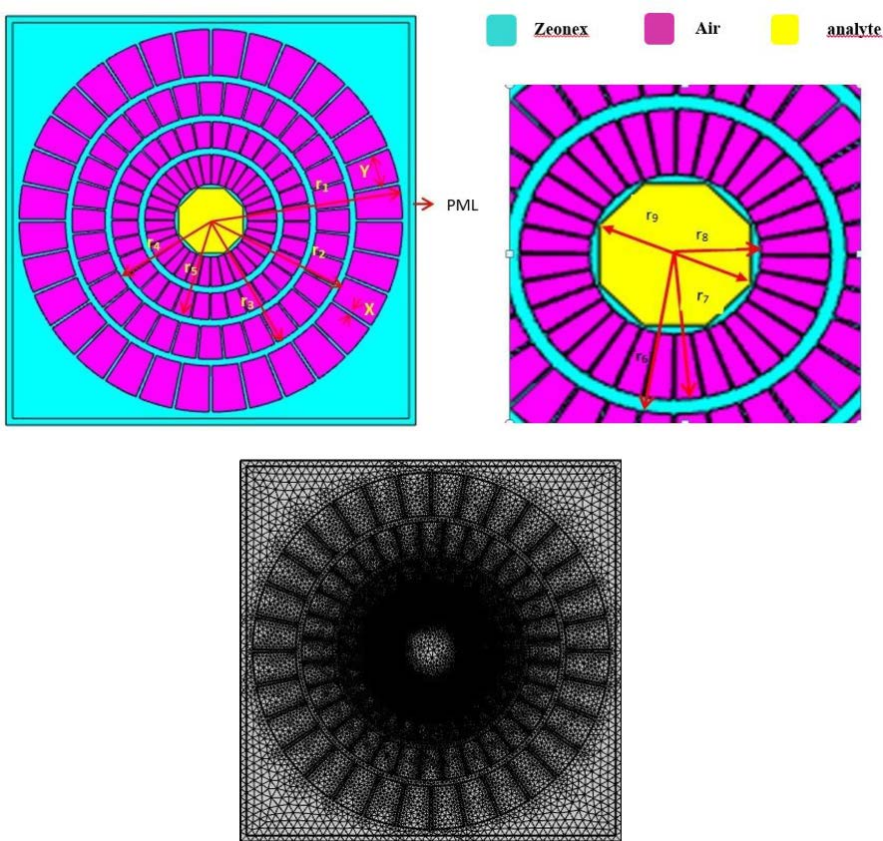
(a) Display recommended PCF in section. (b) The existing mesh style for anticipated PCF.

The complex model of the organisms that live in water biosensor that was described is illustrated in the initial design, which includes every structural detail. THz-founded PCFs typically employ Topas, Zeonex, and Teflon, and substrates made of polymethyl methacrylate (PMMA) due to their exceptional qualities and minimal utilisation losses, compared to other corrective lenses [37]. Zeonex, having the highest see-through ability, the lowest absorption loss, and the strongest strength under stress, is used to make the components surrounding the sensor [38]. With a RI of 1.53, the material is used in the cladding, and PML sections of the device to accomplish its impressive RS and tiny CL. Because of its superior properties and lower transmission losses, Zeonex is the preferred substrate for THz-based PCFs over Teflon, TOPAS, and PMMA. The sensor’s enclosure is made of Zeonex, an outstanding tensile strength, transparent polymer with low absorption loss. Zeonex has various benefits, such as its little optical attenuation, amazing clarity, and ability to interact with living things, which makes it ideal for use in chemical sensing applications with the recommended detector. When compared to alternatives like silica or PMMA, this substance does have certain drawbacks that should be considered. These include the possibility of manufacturing issues and the material’s restricted capacity to change its characteristics. It is assumed that the waterborne bacteria specified in this context have a RI value of 1.333, 1.365, 1.383, or 1.388. Fig 1a illustrates the overview of the planned PCF diagram. A detailed depiction of a circular core structure is depicted in Fig 1a.

**Fig 2:**
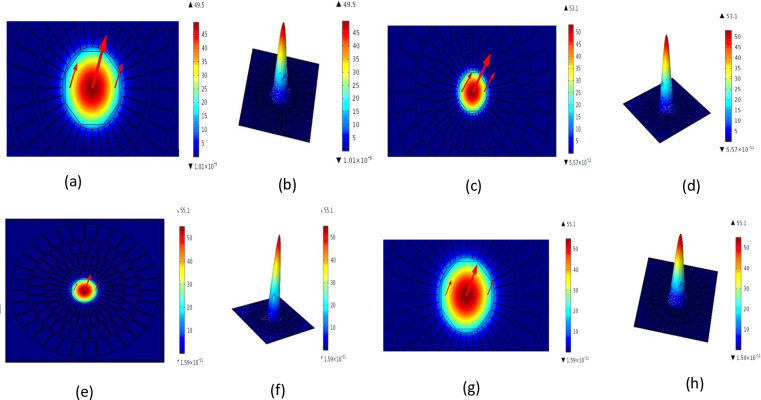
Indicative distribution power and density characteristics.

This pattern which are given in Table 2 consists of three tiers of air openings. On the x-axis, each air opening is separated by a distance of 1 degree, with a full circle radius of 10.25 degrees from the center. It is possible to rotate two rows of air openings by six degrees. PML, or Perfectly Matched Layer, is located on the fiber’s outermost layer. The cladding’s primary function, as perceived from the exterior of the building, is to prevent any light from escaping from the interior of the structure. After successfully reducing back-reflection through the implementation of this technology, the fiber’s overall accuracy is enhanced significantly. The optimal functionality of this surface is contingent upon its alignment, which suggests that it was intended to be integrated into the fiber structure. The material utilized in PCF detector devices is mesh, which is a complex structure that is specifically designed to accommodate optical and recognition functionalities. External element monitoring and beam guidance are both directly influenced by the transparency of the PCF, which is significantly regulated by the mesh pattern. The ventilation spaces of a lattice can be reorganized to optimize the sensitivity of a fiber to sensors and its acoustic trait variation. Fig 1b illustrates the open lattice structure. There are 24174 vertices, 532 vertex pieces, 6444 boundary variables, and 48170 domain parts in the mesh. Fig 2 displays the dispersion of authority and the relationships between various characteristics. By extending the relationship between visible light and matter and the analyte, the spiral-shaped design of a PCF sensor enhances its sensitivity. Light confinement within the core is improved, resulting in a reduction in losses and an improvement in signal quality. Stability is the capacity of the PCF sensor to function reliably under varying circumstances. It guarantees that the sensor produces consistent results throughout time. Reliability indicates that the sensor will function as intended for extended periods of time without experiencing frequent problems. The sensor can withstand damage from harsh conditions, such as contaminated water or extremely high or low temperatures, thanks to its durability. Accurate detection of aquatic bacteria is ensured by these parameters taken together. They enable the sensor to continue working in various scenarios. For long-term water quality monitoring, PCF sensors are ultimately dependable due to their stability, dependability, and longevity. The spiral structure is an optimal choice for high-precision sensing applications due to its ability to fine-tune optimal design parameters with greater flexibility. The distribution of power is determined by the manner in which light transmits through the fiber’s core and cladding. Parameters that affect the variability include the geometry, refractive index, and power coupling factor feeding circumstances. Knowing the direction in which luminosity increases in relation to auditory interactions can help you predict its occurrence with greater accuracy. For the purpose of enhancing the adaptability and effectiveness of robots, ongoing research is being conducted on the reliable detection of changes induced by external variables such as temperature and material composition and energy flow. New methods that enhance precision, reduce error rates, and increase efficiency may result from this data. Compared to a PCF sensor, the density distribution indicates the degree of scattering of a substance. This section demonstrates the impact of the material’s inner and outermost components, as well as its functional fibres, on its rigidity. It is necessary to have a comprehensive understanding of granular dispersion in order to employ monitoring and communication applications. This data is essential for the development of PCF that is precision-accountable, has specific directional qualities, and is adaptable to external factors. THz waves interact with bacterial cells by altering their structure, molecular vibrations, and water content. Certain bacteria, such as *E. coli*, *Vibrio cholera*, and *Bacillus anthracis*, have distinct features that affect how they react to THz waves. Because *Vibrio cholera* contains water, its lipopolysaccharides have an impact on THz wave absorption. *E. coli* exhibits particular absorption due to its DNA and proteins. The thick cell wall and spores of *Bacillus anthracis* result in distinct patterns of absorption and scattering. Various variations enable the sensor to recognise and differentiate various microorganisms in water by detecting them as variations in absorption peaks, scattering, and refractive index.

**Table 1:** Generating geometry parameters for the optimal pitch.

Parameter Name	Relationship with pitch	Actual value for pitch 210 µm
r_1_	2.7985*p	587.685 µm
r_2_	2.123*p	445.83 µm
r_3_	2.0265*p	425.565 µm
r_4_	1.544*p	324.24 µm
r_5_	1.4475*p	303.975 µm
r_6_	1.0615*p	222.915 µm
r_7_	0.965*p	202.65 µm
r_8_	0.53075*p	111.4575 µm
r_9_	0.5145*p	108.045 µm
PML	0.193*p	40.53 µm

**Table 2:** The suggested configuration of the gadget has been compared to many iterations.

Citations	Structures	Analyte	Sensitivity (%)	Frequency (TH)	EML(cm^−1^)	CL(dB/m)
PCF [41]	Heptagon clad plus hexagon core	Ethanol	68.48%	1.0	–	2.13 × 10^−09^
		Benzene	69.20%			1.92 × 10^−09^
		Water	66.78%			2.70 × 10^−06^
PCF [42]	Hexagon core plus octagon clad	Ethanol	77.14%	1.0	–	2.26 × 10^−03^
		Benzene	78.06%			3.02 × 10^−06^
		Water	76.11%			2.76 × 10^−06^
PCF [43]	Hexagon clad as well core	Ethanol	81.46%	1.0	–	5.85 × 10^−08^
		Benzene	82.26%			6.04 × 10^−08^
		Water	79.22%			5.85 × 10^−08^
PCF [44]	flower shape core	Camel milk	88.2%	1.8	0.21595	1.01 × 10^−13^
		Cow milk	88.5%		0.21562	2.71 × 10^−13^
		Buffalo milk	88.8%		0.21520	2.04 × 10^−14^
PCF [45]	Square core and air holes	alcohol	79.3%	3-5	–	0
PCF [46]	Kagome pattern plus rectangular air holes	Water	85.6%	1.6	–	4.5 × 10^−09^
		Ethanol	85.7%			1.71 × 10^−09^
		Benzene	75.9%			1.02 × 10^−09^
PCF [47]	Square clad alongside octagon core	Water	90.65%	1.3	0.01368226	1.88 × 10^−08^
		Ethanol	91.93%		0.01468226	2.00 × 10^−08^
		Benzene	93.06%		0.01568226	2.12 × 10^−08^
This PCF	Circular clad with octagon core	Water	95.40%	3.2	0.00705	4.36 × 10^−13^
		*Vibrio Cholera*	96.78%		0.005641	2.095 × 10^−13^
		*Bacillus anthracis*	97.40%		0.00498	1.355 × 10^−11^
		*E. coli*	97.54%		0.00483	4.411 × 10^−11^

## 3. Experimental evaluations

During the development and research of the features of the proposed sensor, several critical numerical formulas were taken into account. Performance of hollow-core PCF sensors is significantly influenced by criteria, such as RS, CL, and EML, Effective Area, Spot size and Numerical Aperture. These variables impact the degree of light containment, the efficacy of light interaction with the analyte, and the loss that is attributed to the fiber’s material properties. Their presence directly impacts the detector’s ability to identify small modifications within the analyte. Upon collective examination, these parameters serve to enhance the detector’s sensitivity, reliability, and accuracy rendering it suitable for a diverse array of applications that involve detecting.

### 3.1. Empirical formulas

The mathematical equations that are taken into account during the development and analysis of specific proposed sensor attributes are addressed in this section. The formula for determining the RS of any detector is as follows [39].


$$r=\frac{n_{r}}{n_{eff}}\times p\%\;$$ (1)

Therefore, with the exception of the *n*_*eff*_, or supervised option RI, and the *p*, that has the potential to determined through subsequent procedure [39].


$$p=\frac{\int_{sample}R_{e}\left(E_{x}H_{y}-E_{y}H_{x}\right)dxdy}{\int_{total}R_{e}\left(E_{x}H_{y}-E_{y}H_{x}\right)dxdy}$$ (2)

Horizontal magnets In such design, the parallel electric fields are denoted as *E*_*x*_ and *E*_*y*_, while the fundamental aided selection is represented by *H*_*x*_ and *H*_*y*_. The resolution of any detector can be determined using the subsequent formula [39].


$$Aff=\frac{V_{air}}{V_{total}}\times 100$$ (3)

The EML articulation is determined by the formula that follows [39].


$$\alpha_{eff}=\frac{{\left(\frac{\varepsilon_{0}}{\mu_{0}}\right)}^{\frac{1}{2}}\;\int_{A_{\max}}n\alpha_{mat}{\left|E\right|}^{2}dA}{2\int_{ALL}S_{z}dA}$$ (4)

Where αmat alongside *E* are zeonex loss coefficient as well as electric field, consequently.

During monitoring along with connectivity, PCF structures must sustain a low CL to optimise space and enhance overall performance. Utilise the equation below to compute CL [39].


$$L_{c}=\frac{40\pi }{\ln\left(10\right)\lambda }img\left(n_{eff}\right)\times \frac{10^{6}dB}{m}$$ (5)

The imaginary component of EML utilized in this study is *img n*_*eff*_, with its operational wavelength denoted by solving the equation will get the NA of this optical detector [39].


$$NA=\frac{1}{\sqrt{1+\frac{\pi A_{eff}f^{2}}{c^{2}}}}\approx \frac{1}{\sqrt{1+\frac{\pi A_{eff}}{\lambda^{2}}}}$$ (6)

Understanding EA in PCF is essential for the design and optimisation of PCF-materialized optical devices to acquire various applications. To determine EA, employ the formulas provided earlier [40].


$$A_{eff}=\;\frac{{\left[\int I\left(r\right)rdr\right]}^{2}}{{\left[\int I^{2}\left(r\right)rdr\right]}^{2}}$$ (7)

In the domain of electrical sensors, *I*(*r*) = |E|^²^ represents the distribution symbol. The effective area of a PCF detector directly influences its sensitivity and detection capability.

A significant spot dimension is essential to obtain detection, as illustrated by the formula below [40].


$$\;\;W_{eff}=R\times \left(.65\times 1.619\times V^{-1.5}+2.789\times V^{-6}\right)$$ (8)

where *R* denotes circumference of the hexagonal core, with *V* standing for the normalized frequency value.

### 3.2. Experimental setup

**Fig 3:**
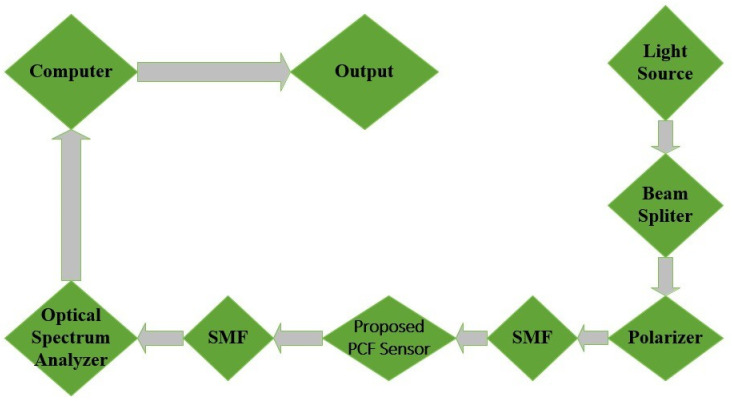
Experimental configuration for the proposed sensor.

A terahertz light source is employed to produce a specific wavelength that is appropriate for the purpose of identifying microorganisms in water in the setup which is shown in Fig 3. By dividing the light, the beam splitter enables a portion of it to interact with the bacterium sample in the suggested PCF sensor. This sensor is intended to improve the interaction between bacterial cells and terahertz waves. The sensor’s sensitivity is optimised by the polariser, which guarantees that only light with a specific polarisation entered the device.

The optic properties about terahertz light, encompassing its index of refraction, absorption, and transmission spectrum, are influenced by the presence of microbes in the water as it passes through the PCF sensor. It is the optical spectrum analyser that detects variations in the terahertz signal that captures these changes. This data is subsequently processed by the computer, which then correlates the observed spectral changes to bacterial concentrations. This process enables the precise detection and real-time monitoring of waterborne pathogens.

This approach capitalizes on the high sensitivity of terahertz waves to molecular vibrations, rendering it optimal for the identification of biological entities such as microorganisms.

## 4. Results analyses

In this discipline, the phrase “Finite Element Method (FEM)” denotes the procedure through dividing the PCF design into more fundamental elements. Each object’s refractive characteristics are evaluated by employing geometrical concepts alongside the index of refraction. The development of sensing devices based on Photonic Crystal Fiber (PCF) relies heavily on the use of COMSOL Multiphysics 6.1 and the Finite Element Method (FEM) for accurate optical property optimization and simulation. The complex microstructures of PCFs may be accurately modelled using COMSOL, enabling researchers to examine their light interaction in great detail. The program numerically solves Maxwell’s equations using finite element method (FEM), which provides information on the PCF’s field distribution, effective refractive index, confinement losses, and mode propagation. This computational method allows for the optimization of parameters including core size, air-hole configuration, and material properties without the need for time-consuming and expensive experimental trials which helps in the construction of extremely sensitive and selective sensors. As a result, this paves the way for the efficient and accurate creation of specialized PCF sensors for uses including waterborne bacteria detection and environmental monitoring.

By solving the relevant equations with the FEM, the ultrasound Characteristics and the geographical dispersion of light can be ascertained. Therefore, propagation of light can be replicated by employing PCF. This approach helps to assess intricate PCF designs and improves their dependability for a variety of optoelectronic applications. A multitude of factors that affect the manipulation and transmission of light in complex PCF systems are examined using the FEM. It is essential to possess this information in order to enhance and construct instruments that are based on PCF. We utilized a frequency range of 2.6 THz to 3.8 THz to find out waterborne *Vibrio cholera*, *E. coli*, and *Bacillus anthracis* in the water in this simulation. The the indexes of refraction of the pathogens are 1.365, 1.383, 1.388 and so on. We will consistently obtain the highest RS and lowest CL by adjusting the pitch within the 180 micrometer to 240 micrometer range for this project. It is essential to consider the following aspects in the pursuit of a thorough comprehension of the sensory qualities CL, RS, NA, EML, and aff.

Sensitivity is the measure of a detector’s ability to detect minor fluctuations in variables such as bacterial concentration, detection limit, sample inhibitory chemicals, amplification efficiency. As a result of the fiber’s extreme sensitivity, it is feasible to conduct more accurate and reliable measurements of light transmission, even in the presence of minute environmental fluctuations. The microstructure of the PCF can be altered to enhance sensitivity and intensify light-matter interactions. Typical optical fibers and standard single-mode fibers are both terms that refer to the same thing: transparent, flexible filaments of Glass or acrylic that convey light information at a low cost. Its core and cladding surface are engineered to effectively transmit light through complete inner reflection, rendering it an optimal choice for data transfer over long distances and at a rapid pace. Response time to environmental disturbances is improved. The optimization of RS is necessary to produce PCF sensors that are affordable, accurate, and suitable for use in environmental monitoring and clinical trials. The layout, setup, and planned use of a PCF detector can influence its RS. Sensors are typically calibrated and characterized by engineers and scientists to ascertain their sensitivity in a variety of environments. Reliable and precise measurements for real-world applications are ensured by this.

Sensitivity of hollow-core PCF waterborne bacteria detection sensors is contingent upon the interaction between pitch and frequency. The frequency is varied at a constant pitch, which initially results in an increase in sensitivity.

**Fig 4:**
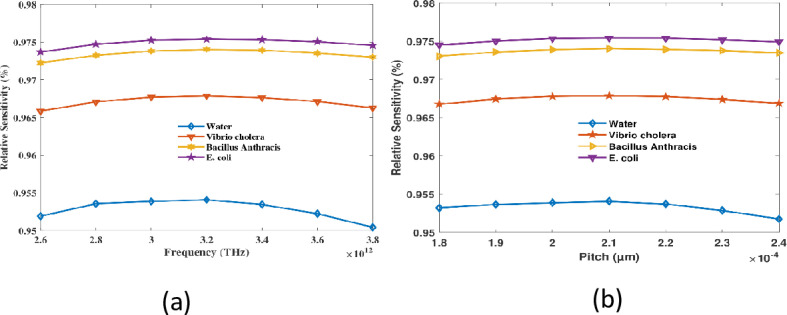
Shows the sensors’ sensitivity to (a) periodicity at a predefined pitch of 320 µm and (b) pitch at 2.1 THz.

Precisely as it does when the pitch is adjusted at a consistent frequency. At higher frequencies, however, it decreases as light passes through the cladding. As illustrated in Fig 4a, an RS plot is the outcome of a frequency variation. At an optimal pitch of 210 µm, sensor RS values of 95.40%, 96.78%, 97.40%, and 97.54% were recorded for RI values of 1.333, 1.365, 1.383, and 1.388, respectively. The RS measures the degree of interaction between the directed light and the analyte and express the effectiveness of the sensor in detecting bacteria. Increases in RS indicate better light-matter interaction, which in turn leads to more precise and accurate bacterial identification. These numbers show how the sensor has been becoming better over time by optimizing its structural parameters like core size and air-hole configuration. The improved sensitivity and robust performance of the sensor are demonstrated by this rise in RS, which is significant even in the presence of low bacterial concentrations. The sensor’s great sensitivity makes it a potential tool for public health, environmental monitoring, and use in places with limited resources, where accurate and efficient bacterial identification is crucial.

According to the curve, the frequency-dependent connect visual intensity to concentrations or specimen culminates in a high point at 3.2 THz

Fig 4b illustrates the RS reaction of the curve to changes in pitch. The effective refractive index and the photonic bandgap are factors that influence the sensitivity when the pitch is variable at a fixed frequency of 3.2 THz. This influences the confinement of light and forms an overlap with the analyte. The optimum value is attained at 210 µm, and the RS remains consistent. The complex sensitivity response is the outcome of the combined effects of these factors in scenarios where neither pitch nor frequency are fixed. This response is contributed to by the delicate equilibrium between coupling efficacy, bandgap adjustment, and mode confinement. Within the strand’s structure, the combined effect of multiple loss pathways is illustrated by EML in PCF. The typical level of spotlight is reduced so that it travels via the PCF due to a variety of processes, including light absorption, light dispersion, and the presence of other substances. The effects of radio frequency interference must be taken into account when evaluating optoelectronic schemes that utilize polymer-coated strands. Data transmission efficiency and system reliability are substantially affected by this. Optimized calibration is essential for the successful elimination of deficiencies in PCF appliances. This can be determined by measuring the decrease in luminance as a function of vertical distance. Competence in EML is required to enable the modification of PCF properties for the purpose of sensing and internet access. When transmission results are optimal, it is particularly advantageous to employ efficient methods that minimize signal loss.

**Fig 5:**
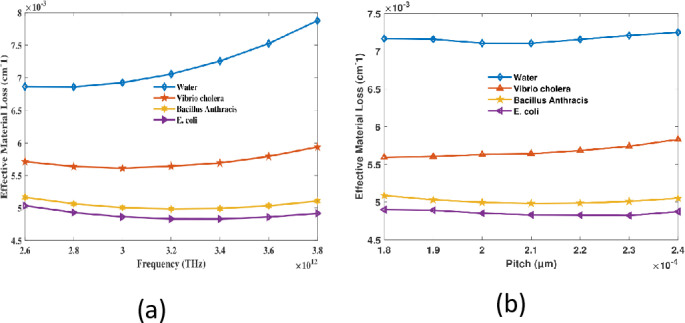
EML of the proposed sensor in regard to (a) harmonics at a specified pitch of 210 µm and (b) pitch at 3.2 THz.

The EML of hollow-core PCF waterborne bacteria detectors is variable in response to frequency as a result of the interaction between the fiber material and focused light. When operating at lower frequencies, the core is more adept at retaining the light, which leads to a reduction in material contact and a decrease in loss (EML). The intense light-material interactions at higher frequencies that are induced by mode coupling are the cause of elevated EML. Changes in the photonic bandgap alter the efficacy of light confinement within the core, which in turn affects the EML behavior when the pitch is altered at a fixed frequency. Variations in pitch affect the bandgap that have an impact on the amount of radiance that reaches cladding. As a result, EML is exposed to these fluctuations. EML’s non-linear behavior in both instances may be ascribed towards the equilibrium in between light confinement along mode- substance interaction within the fiber. RI 1.333, 1.365, 1.383, and 1.388 have EMLs of 0.00705 cm^−1^, 0.005641 cm^−1^, 0.00498 cm^−1^, and 0.00483 cm^−1^, respectively, when the pitch is 210 µm and the frequency is 3.2 THz which is depicted in Fig 5a,b. The EML of the sensor is exceptionally low, rendering it an ideal choice for energy-efficient, high-performance applications.

Cl measures the amount of light that is lost during its passage through a PCF and into the surrounding substance or packaging. This matter is predominantly result of inadequate luminescent confinement in middle. Most frequently, that is the result of structural discrepancies or errors in PCF structure. The consideration of CL is essential for PCF to operate on optical devices, particularly in circumstances at which minimal damages and accurate ray guidance are essential. Order to quantify phase leakage, the quantity of luminous that leaks out of every single optical fiber is quantified. To achieve optimal monitoring and connection performance, PCF structures must be space-efficient and generally minimal in CL. PCF sensors with extremely low cladding losses enhance signal retention, guaranteeing more robust and distinct signals for pathogen detection. By doing this, the sensor’s sensitivity is increased and it can identify pathogens at lower concentrations. The sensor’s detection range is further increased by the decreased energy loss, making it useful over greater water volumes. Because there is less cladding loss, the sensor operates steadily and consistently and needs less recalibration. They also use less power because these sensors are more energy-efficient. This makes it possible to detect pathogens with accuracy even in difficult or murky real-world water sources.

PCF structures must maintain a low CL in order to achieve optimal monitoring and linking performance, while also conserving space and being generally efficient.

**Fig 6:**
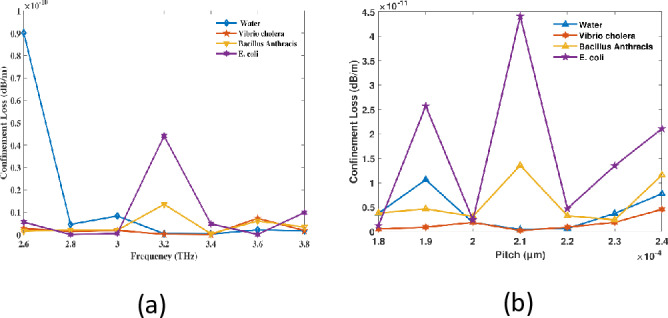
The CL of developed tracker with respect to (a) resonance at an organized pitch of 210 µm and (b) pitch at 3.2 THz.

**Fig 7:**
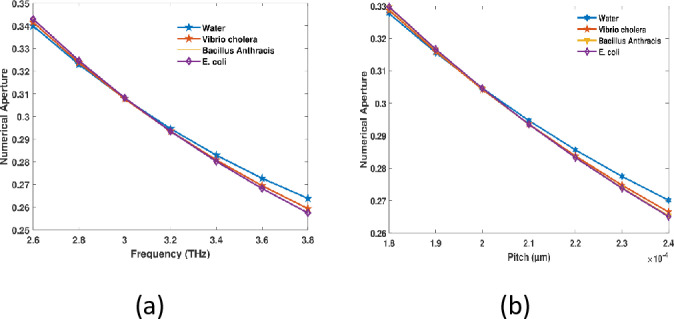
The advised actuators NA for (a) frequency at 210 µm preset pitch and (b) pitch at 3.2 THz.

Due to fluctuations in of PCF sensors enhances, the CL reduces, while pitch remains constant. This is due to the increased losses that arise from the diminished confinement at lower frequencies. In a steady state, the confinement is not substantially impacted by further frequency increases; at this juncture, the loss is negligible as the mode becomes more tightly constrained at the center with rising frequencies. The periodic fluctuation of photonic bandgap is the cause of this. These shifts are responsible for the fluctuating behavior of the confinement loss, which leads to the light experiencing varying levels of confinement as the pitch changes. This implies that the cladding is emitting varying amounts of light, which is conditional upon the specific pitch values. At a spectrum 3.2 THz with a pitch of 210 µm, the CL for RI values of 1.333, 1.365, 1.383, and 1.388 is 4.36 × 10^−13^ dB/m, 2.095 × 10^−13^ dB/m, 1.355 × 10^−11^ dB/m, and 4.411 × 10^−11^ dB/m, respectively, as illustrated in Fig 6. As frequency and pitch increase in hollow core PCF indicators, the fundamental radiant propagation characteristics of photonic crystal structures can be attributed to the reduction in numerical aperture (NA).The wavelength decreases as the frequency increases, which results in a reduced contrast in effective index of refractive between cores along with cladding. The result is a decrease in NA due to the weakening of light confinement. Similarly, the index contrast diminishes as the pitch (distance between air apertures) increases, which ultimately results in a lower NA and further weakens light confinement. Therefore, the capacity of the fiber to efficiently the navigation illumination is diminished as a consequence of the increased frequency and larger pitch, which leads to a decrease in NA. As the effective RI contrast positioned within the structure and cladding decreases, the fiber’s light acquisition capacity is reduced. As the pitch rises, this occurrence transpires at a consistent frequency of 3.2 THz. For RI 1.333, 1.365, 1.383, and 1.388, the NA values are 0.2946 at a regularity of 3.2 THz and a pitch of 210 µm. Conversely, they are 0.2935 for RI 0.2934 and 0.9359 which are demonstrated in Fig 7. The numerical aperture (NA) values are essential to the PCF sensor’s efficient operation since they establish its capacity to collect light and its confinement efficiency. Better light collecting from external sources and stronger light confinement within the fiber core are indicated by higher NA values, such as 0.9359, which improve the interaction between the analyte and the guided modes. In order to identify germs with high sensitivity, this interaction is essential. On the other hand, somewhat lower NA values between 0.2934 and 0.2946 indicate a balance between dispersion management and light guidance, guaranteeing reliable optical mode propagation. By facilitating accurate mode control, excellent analyte-light interaction, and flexibility to meet changing operational needs, these NA values collectively maximise the sensor’s performance and increase its dependability and efficacy in bacterial detection.

**Fig 8:**
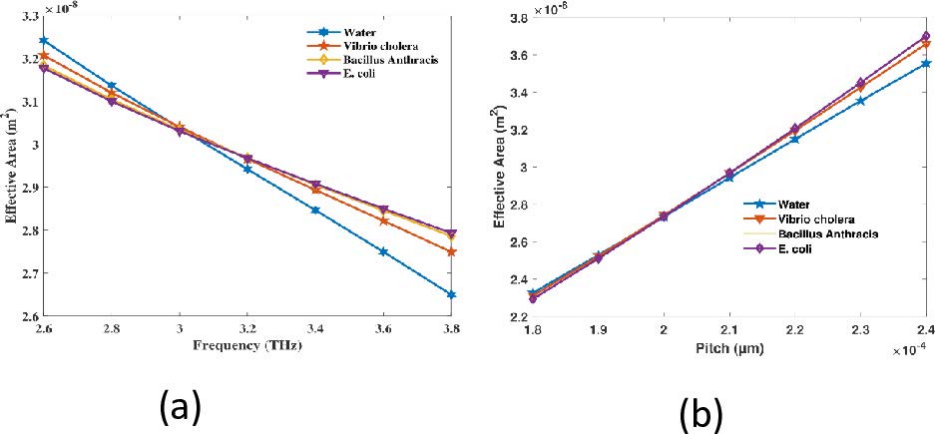
An evaluation of the proposed sensor in regard to (a) periodicity with an established pitch of 210 µm and (b) pitch at 3.2 THz.

Variations in effective area of the PCF sensor are likely due to luminescent encirclement properties of fiber with respect to frequency and pitch. The wavelength shortens, which increases containing light inside the central. Consequently, a lower effective area is observed with higher frequencies from 2.6 to 3.8 THz which is shown in Fig 8a. This resulted in a reduced effective area and a more concentrated light field. On the other hand, the effective area increases while the pitch rises from 180 to 240 μm which is depicted in Fig 8b. This is the result of the larger spacing between the air openings, which reduces the refractive index contrast, thereby weakening light confinement and allowing the light to disperse over a broader space.

As a consequence of the dispersion of light within the core, the EA increases due to the broader mode distribution, which is less concentrated. At a resonance of 3.2 THz and a pitch of 210 µm, the EA is 2.942 × 10^8^ m^2^, 2.966 × 10^8^ m^2^, 2.968 × 10^8^ m^2^, and 2.9681 × 10^8^ m^2^ for RI values of 1.333, 1.365, 1.383, and 1.388, respectively.

**Fig 9:**
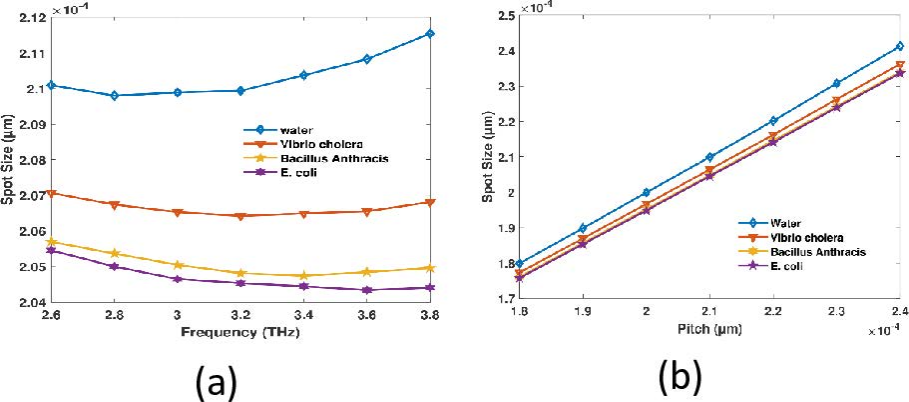
Potential sensor spot size in regards to (a) frequency at a prearranged pitch of 210 µm and (b) pitch at 3.2 THz.

In a photonic crystal fiber (PCF) sensor, the spot size trends can be attributed to the influence of pitch and frequency on light propagation. Typically, the tighter confinement of light within the core results in a reduced spot size as the frequency grows from 2.6 to 3.8 THz which is illustrated in Fig 9a, as the wavelength of light decreases. The extent of the spot diminishes as the pitch expands from 1.8 to 2.4 μm which is shown in Fig 9b. Larger pitch values can enhance light confinement by increasing the photonic bandgap effect, which leads to a more focused light pinpoint. This is the reason. Both effects reduce the spot size, albeit through distinct mechanisms that are linked to wavelength and structural parameters.

As the frequency increases to 3.2 THz, the spot size of PCF pathogens detection sensors is diminished therefore of the manner being greater restricted inside the heart. As a consequence of mode leakage into the cladding or higher-order mode effects, the confinement effectiveness begins to diminish at frequencies exceeding 3.2 THz, and the spot size is progressively increased. RI 1.333, 1.365, 1.383, and 1.388 have spot diameters of 2.099 × 10^−04^ µm, 2.0641 × 10^−04^ µm, 2.0481 × 10^−04^ µm, and 2.045 × 10^−04^ µm, correspondingly at a frequency of 3.2 THz and a pitch of 210 µm.

A systematic comparison was conducted between the proposed sensor and existing PCF sensors. Table 2 summarizes the primary attributes of the current and of the PCF sensors that we have investigated. Tailoring the precursor material to the specific use case is necessary for the production of PCFs. For the prototype, silicon dioxide or a comparable substance is typically employed. One of the most prevalent fabrication methods is the stacking and drawing process, which entails the heating of the preform and its subsequent reduction to a smaller fiber while maintaining its original structure. In order to create distinctive characteristics and establish communication pathways between analytes, procedures such as drilling perforations or etching are implemented [39,40].Covering the sensor’s surface with functional materials can enhance its sensitivity and selectivity. It’s crucial to take cost, scalability, and manufacturing viability into account when designing PCF sensors for large-scale applications to ensure their efficient production and utilization [48,49]. Both producers and users can afford the sensors thanks to their inexpensive pricing, particularly in areas with limited resources. Scalability guarantees the sensors’ capacity to be produced in big quantities without compromising quality or raising prices. Manufacturing viability guarantees that the sensors can be manufactured effectively and consistently, enabling uniform performance in each unit. Resolving these issues contributes to the widespread adoption and viability of PCF sensors for large-scale applications. Optimize the fiber design for improved sensitivity and specificity while utilising inexpensive, high-performance materials to produce PCF sensors that are affordable. To improve bacterial detection and incorporate effective detection technologies, use surface alterations. Miniaturisation and automated manufacturing can lower production costs. Make sure the sensors are easy to repair and long-lasting to reduce long-term costs. Lastly, carry out field testing to improve the sensor’s functionality and make sure it satisfies practical needs. There are a number of obstacles that must be overcome before PCF sensor technology may be applied in commercial settings. To be successful, the sensors need to be mass-produced in a way that is both sturdy and inexpensive. Additionally, they should be compatible with current monitoring systems and easy for humans to utilise. The sensors, in order to be used in environmental and health monitoring in the actual world, need to be safe and of high quality. Finding solutions to these problems will make PCF sensors more practical for usage in everyday life. In the detection of waterborne bacteria, the efficiency of this PCF sensor is impacted by numerous variables, including the operating wavelength for optimal light-bacteria interaction, fiber design (core geometry and microstructure), and surface functionalization for specific bacterial binding. Moreover, pH and temperature are environmental variables that should be taken into account. The sensor is capable of detecting waterborne bacteria with precision by fine-tuning parameters such as pitch and frequency, which result in high sensitivity and minimal losses. The timely and effective surveillance of bacterial contamination in water sources is contingent upon accuracy, which is essential for the protection of public health and safety. In addition to, this PCF sensor provides a dependable and extremely sensitive way to identify dangerous bacteria and viruses in water, which enhances water quality monitoring and public health protection. Ideal for early contamination detection, it allows faster actions to avoid waterborne infections by detecting low quantities of bacteria like E.coli and other waterborne pathogens. Continuous assessment of water quality is made possible by the PCF sensor, which reduces the danger of public health hazards due to its high sensitivity, low detection limits, and capability for real-time monitoring. In addition to helping to make drinking water cleaner and better protecting public health, its compact size, low cost, and user-friendliness make it ideal for broad deployment in both resource-rich and resource-constrained areas. The accuracy and efficacy of bacteria detection are enhanced, thereby reducing the risk of waterborne diseases and improving water quality management, by optimizing these critical parameters in PCF sensors.

## 5. Comparison with Other PCF

The PCF built for detecting aquatic bacteria outperforms the other PCFs shown in Table 2. It outperforms other PCFs in terms of sensitivity, with Relative Sensitivity (RS) values of 95.40% for water, 96.78% for *Vibrio cholera*, 97.40% for *Bacillus anthracis*, and 97.54% for *E. coli*. In contrast, the greatest RS recorded in the other designs is around 93.06% (benzene in PCF [43]). This suggests a significant improvement in target analyte detection, particularly in biological and environmental situations.This bacteria-detecting PCF has lower Confinement Loss (CL) values, ranging from 4.36 × 10^−13^ to 4.411 × 10^−11^ dB/m compared to other PCFs that exhibit confinement losses. Ensuring that the bulk of the light energy stays confined within the fiber core, the low Confinement Loss (CL) values (ranging from 4.36 × 10^−13^ to 4.411 × 10^−11^) greatly improve the PCF sensor’s detection accuracy. A more sensitive and reliable detection method is achieved when there is little light loss because the guided modes interact with the analyte more efficiently. Reducing noise and enhancing sensor resolution are two additional benefits of low CL values, which help keep signals intact during extended interaction periods. For low-concentration bacterial detection, this property is crucial, since it permits accurate readings with little optical information loss, guaranteeing the sensor’s usefulness in practical settings. The rapid identification of pathogens is enhanced by the high sensitivity of PCF, which enables improved detection of aquatic bacteria. When the confinement loss is low, the signal attenuation is minimal, and the light interacts with the sample more effectively, which improves the measurement accuracy and reliability. All of these characteristics work together to make PCF-based sensors superior to conventional approaches for environmental monitoring, allowing for faster and more accurate detection. A PCF sensor with exceptional sensitivity, robust stability, selectivity, low production costs, extended expiration life, and suitability for widespread industrial use is expected to be developed in the future. Ongoing research innovations and advancements in technology and processing techniques will be the driving force behind this development. The PCF-based THz sensor’s current drawbacks include its low sensitivity at extremely low bacterial concentrations, its difficulty differentiating between closely related species, and its susceptibility to interference from environmental elements like turbidity, salinity, and temperature. By improving the fiber structure and incorporating more sensitive detecting techniques, future advancements might concentrate on increasing sensitivity. Surface functionalisation to bind specific bacteria or sensor refinement to concentrate on distinct bacterial signals are two ways to increase specificity. Accuracy could be increased by using protective coatings or compensatory algorithms to address environmental interference. Additionally, the sensor would be more portable for real-world applications if it were smaller.

## 6. Conclusion

Using sophisticated sensing techniques is a crucial first step in identifying aquatic bacteria. The non-invasiveness, ease of detection, simplicity of sample collection, and high patient and environmental applicability of aquatic bacteria detection are some of the benefits offered by this technology over older methods. Hence, that is widely used to accomplish the goal of water quality observe and pathogen detection. In order to detect different amounts of bacteria in water, we used COMSOL Multiphysics 6.1 and the Method of Finite Element to construct and test a PCF sensing device. Improving water quality monitoring and public health safety, this PCF sensor provides a viable method for precise and efficient bacterial identification. Based on the parameters supplied, the PCF sensor, which is intended to detect waterborne bacteria, exhibits impressive performance. A high degree of sensitivity to bacterial concentrations in water is indicated by the Relative Sensitivity (RS) values of 95.40%, 96.78%, 97.40%, and 97.54%. The detection accuracy is improved by the minimal signal degradation, which is ensured by the low Confinement Loss (CL) values, which range from 4.36 × 10^−13^ dB/m to 4.411 × 10^−11^ dB/m.In addition, the sensor’s ability to operate effectively capture and direct light is demonstrated by the NA values of 0.2946, 0.2935, 0.2934, and 0.9359. This makes the PCF sensor an extremely promising solution for environmental and health surveillance, as it is highly suitable for the precise and dependable identification of microorganisms in water. The transformation of watery germs detection from research settings to practical applications necessitates the creation of cost-effective Photonic Crystal Fiber (PCF) sensors that deliver precise and accurate qualitative results. To ensure that water quality monitoring is effective, these sensors must meet the rigorous standards of sensitivity and specificity by demonstrating high stability, dependability, and durability. In addition to addressing these technical challenges and undertaking comprehensive field evaluations, it is essential to take into account practical factors such as cost, scalability, manufacturing feasibility, and user-friendliness when developing new, large-scale solutions for waterborne bacteria detection.

## Supporting information

S1 Data f final. (PDF)

S2 Data p final. (PDF)
