# Supplementary material for: Optimized PCF architectures for THz detection of aquatic pathogens: Enhancing water quality monitoring
Source: PLoS One. 2025 Jan 27;20(1):e0317533. doi: 10.1371/journal.pone.0317533 (PMC11771926; doi:10.1371/journal.pone.0317533)
Supplement: S1 Data — (PDF) [file pone.0317533.s001.pdf]

wave	freq	p	nr	l	aff	real-x pol	real-y p
1.15E-04	2.60E+12	2.10E-04	1.388	0.000327	0.965		1.34860000000000
1.07E-04	2.80E+12	2.10E-04	1.388	0.000327	0.965		1.35370000000000
1.00E-04	3.00E+12	2.10E-04	1.388	0.000327	0.965		1.35790000000000
9.38E-05	3.20E+12	2.10E-04	1.388	0.000327	0.965		1.36150000000000
8.82E-05	3.40E+12	2.10E-04	1.388	0.000327	0.965		1.36450000000000
8.33E-05	3.60E+12	2.10E-04	1.388	0.000327	0.965		1.36700000000000
7.89E-05	3.80E+12	2.10E-04	1.388	0.000327	0.965		1.36920000000000
1.15E-04	2.60E+12	2.10E-04	1.365	0.000327	0.965		1.32650000000000
1.07E-04	2.80E+12	2.10E-04	1.365	0.000327	0.965		1.33160000000000
1.00E-04	3.00E+12	2.10E-04	1.365	0.000327	0.965		1.33580000000000
9.38E-05	3.20E+12	2.10E-04	1.365	0.000327	0.965		1.33930000000000
8.82E-05	3.40E+12	2.10E-04	1.365	0.000327	0.965		1.34230000000000
8.33E-05	3.60E+12	2.10E-04	1.365	0.000327	0.965		1.34480000000000
7.89E-05	3.80E+12	2.10E-04	1.365	0.000327	0.965		1.34700000000000
#DIV/0!							
#DIV/0!							
1.15E-04	2.60E+12	2.10E-04	1.333	0.000327	0.965		1.29630000000000
1.07E-04	2.80E+12	2.10E-04	1.333	0.000327	0.965		1.30120000000000
1.00E-04	3.00E+12	2.10E-04	1.333	0.000327	0.965		1.30540000000000
9.38E-05	3.20E+12	2.10E-04	1.333	0.000327	0.965		1.30880000000000
8.82E-05	3.40E+12	2.10E-04	1.333	0.000327	0.965		1.31180000000000
8.33E-05	3.60E+12	2.10E-04	1.333	0.000327	0.965		1.31430000000000
7.89E-05	3.80E+12	2.10E-04	1.333	0.000327	0.965		1.31650000000000
#DIV/0!							
1.15E-04	2.60E+12	2.10E-04	1.3833	0.000327	0.965		1.34400000000000
1.07E-04	2.80E+12	2.10E-04	1.3833	0.000327	0.965		1.34920000000000
1.00E-04	3.00E+12	2.10E-04	1.3833	0.000327	0.965		1.35340000000000
9.38E-05	3.20E+12	2.10E-04	1.3833	0.000327	0.965		1.35690000000000
8.82E-05	3.40E+12	2.10E-04	1.3833	0.000327	0.965		1.35990000000000
8.33E-05	3.60E+12	2.10E-04	1.3833	0.000327	0.965		1.36250000000000
7.89E-05	3.80E+12	2.10E-04	1.3833	0.000327	0.965		1.36470000000000

imag-x pol	imag-	area-x pol	are	power-x pol	powe
1.1843000000000E-17		3.1780000000000E-08		9.4606000000000E-01	
1.5931000000000E-19		3.1005000000000E-08		9.5064000000000E-01	
9.2812000000000E-19		3.0315000000000E-08		9.5412000000000E-01	
7.5780000000000E-17		2.9681000000000E-08		9.5679000000000E-01	
7.8211000000000E-18		2.9085000000000E-08		9.5882000000000E-01	
2.1699000000000E-19		2.8509000000000E-08		9.6032000000000E-01	
1.4290000000000E-17		2.7939000000000E-08		9.6138000000000E-01	
5.8114000000000E-18		3.2079000000000E-08		9.3861000000000E-01	
2.9753000000000E-18		3.1207000000000E-08		9.4342000000000E-01	
3.5409000000000E-18		3.0412000000000E-08		9.4701000000000E-01	
3.5996000000000E-19		2.9664000000000E-08		9.4965000000000E-01	
1.1864000000000E-19		2.8942000000000E-08		9.5155000000000E-01	
1.1169000000000E-17		2.8225000000000E-08		9.5282000000000E-01	
2.6439000000000E-18		2.7499000000000E-08		9.5354000000000E-01	
1.9056000000000E-16		3.2424000000000E-08		9.2570000000000E-01	
8.3667000000000E-18		3.1373000000000E-08		9.3082000000000E-01	
1.5356000000000E-17		3.0384000000000E-08		9.3413000000000E-01	
7.4911000000000E-19		2.9425000000000E-08		9.3676000000000E-01	
5.5271000000000E-19		2.8471000000000E-08		9.3830000000000E-01	
3.2813000000000E-18		2.7502000000000E-08		9.3888000000000E-01	
2.5463000000000E-18		2.6504000000000E-08		9.3867000000000E-01	
3.4780000000000E-18		3.1843000000000E-08		9.4464000000000E-01	
4.1471000000000E-18		3.1051000000000E-08		9.4927000000000E-01	
3.6771000000000E-18		3.0341000000000E-08		9.5278000000000E-01	
2.3291000000000E-17		2.9687000000000E-08		9.5545000000000E-01	
7.3221000000000E-19		2.9067000000000E-08		9.5746000000000E-01	
9.2912000000000E-18		2.8465000000000E-08		9.5892000000000E-01	
4.9658000000000E-18		2.7867000000000E-08		9.5994000000000E-01	

sens-x pol	sens y-p	EML-x pol	EML-y po	confinement -x pol
0.9736995995848		0.0050347000000		5.601560273818E-12
0.9747272807860		0.0049290000000		8.114747394505E-14
0.9752695780249		0.0048630000000		5.065231770798E-13
0.9754127947117		0.0048316000000		4.411421093147E-11
0.9753332063027		0.0048308000000		4.837496813947E-12
0.9750725384053		0.0048594000000		1.421072243174E-13
0.9745803680982		0.0049170000000		9.878471735619E-12
0.9658519788918		0.0057105000000		2.748704498460E-12
0.9670834334635		0.0056365000000		1.515523691097E-12
0.9677112217398		0.0056115000000		1.932452611432E-12
0.9678729560218		0.0056412000000		2.095454126008E-13
0.9676419205841		0.0056900000000		7.338106174408E-14
0.9671321386080		0.0057917000000		7.314602462791E-12
0.9662821826281		0.0059361000000		1.827690092499E-12
#DIV/0!				
0.9519078145491		0.0068653000000		9.013200420322E-11
0.9535682908085		0.0068594000000		4.261732284578E-12
0.9538802589245		0.0012484400000		8.380564913199E-12
0.9540808985330		0.0070567000000		4.360833537988E-13
0.9534638664431		0.0072561000000		3.418614854735E-13
0.9522384843643		0.0075269000000		2.148930527456E-12
0.9504345689328		0.0078784000000		1.760220614444E-12
#DIV/0!				
0.9722622857143		0.0051639000000		1.645041512483E-12
0.9732620745627		0.0050636000000		2.112401539122E-12
0.9738292995419		0.0050048000000		2.006784009008E-12
0.9740393433562		0.0049819000000		1.355851262609E-11
0.9739351555261		0.0049916000000		4.528855969288E-13
0.9735589255046		0.0050328000000		6.084827146771E-12
0.9730233765663		0.0051050000000		3.432786210269E-12

confinement-y pol	Total loss-x pol	Total loss-y pol
	5.034700005602E-03	
	4.929000000081E-03	
	4.863000000507E-03	
	4.831600044114E-03	
	4.830800004838E-03	
	4.859400000142E-03	
	4.917000009878E-03	
	5.710500002749E-03	
	5.636500001516E-03	
	5.611500001932E-03	
	5.641200000210E-03	
	5.690000000073E-03	
	5.791700007315E-03	
	5.936100001828E-03	
	6.865300090132E-03	
	6.859400004262E-03	
	1.248440008381E-03	
	7.056700000436E-03	
	7.256100000342E-03	
	7.526900002149E-03	
	7.878400001760E-03	
	5.163900001645E-03	
	5.063600002112E-03	
	5.004800002007E-03	
	4.981900013559E-03	
	4.991600000453E-03	
	5.032800006085E-03	
	5.105000003433E-03	

v para-x pol	NA- x pol	spot-x pol
3.75496630412125E+00	3.43015392177099E-01	2.054545057943E-04
3.77653906390115E+00	3.24697836889851E-01	2.049985716980E-04
3.79337506389241E+00	3.08258119098316E-01	2.046473881748E-04
3.79908229479496E+00	2.93492441877492E-01	2.045292506005E-04
3.80325492161872E+00	2.80205387145627E-01	2.044431678228E-04
3.80848170479950E+00	2.68247929664875E-01	2.043356802121E-04
3.80518328275283E+00	2.57489912227614E-01	2.044034672216E-04
3.68111875906899E+00	3.41600434744156E-01	2.070677423506E-04
3.69588503738928E+00	3.23755750593000E-01	2.067384904104E-04
3.70542320437317E+00	3.07812776475035E-01	2.065276158604E-04
3.71041851136171E+00	2.93569282089788E-01	2.064177363077E-04
3.70713977599659E+00	2.80842300681976E-01	2.064898140092E-04
3.70446698533925E+00	2.69496563124840E-01	2.065486930939E-04
3.69269650792265E+00	2.59404172737437E-01	2.068092984350E-04
3.55226514670906E+00	3.39989349382597E-01	2.100926278384E-04
3.56430523106755E+00	3.22987668964385E-01	2.097979128471E-04
3.56061517838749E+00	3.07941130776697E-01	2.098879616025E-04
3.55866046188214E+00	2.94655993771166E-01	2.099357612605E-04
3.54096952906638E+00	2.82971229963314E-01	2.103714948136E-04
3.52292832805068E+00	2.72755714034551E-01	2.108217365726E-04
3.49451134364663E+00	2.63895408455098E-01	2.115432433187E-04
3.74382037343561E+00	3.42715795369346E-01	2.056927318739E-04
3.75918959629874E+00	3.24482579323925E-01	2.053647159788E-04
3.77441256160898E+00	3.08138559323003E-01	2.050432161123E-04
3.78548907109116E+00	2.93465336204943E-01	2.048113813131E-04
3.78873740587176E+00	2.80285319750558E-01	2.047437239754E-04
3.78396876237100E+00	2.68440244168902E-01	2.048430982406E-04
3.77856899681506E+00	2.57800256217904E-01	2.049560151431E-04

nonlinear- x pol		birefringence
4.729205286344E-05	1.013536952223E+01	1.34860000000000E+00
5.220294533140E-05	9.445472294207E+00	1.35370000000000E+00
5.720478970807E-05	8.840939918439E+00	1.35790000000000E+00
6.232182473636E-05	8.301047935747E+00	1.36150000000000E+00
6.757383393502E-05	7.822227222321E+00	1.36450000000000E+00
7.299434704830E-05	7.396450479426E+00	1.36700000000000E+00
7.862152260281E-05	7.008841903019E+00	1.36920000000000E+00
4.685125596184E-05	1.005802463697E+01	1.32650000000000E+00
5.186504053578E-05	9.367393666809E+00	1.33160000000000E+00
5.702233328949E-05	8.761706526057E+00	1.33580000000000E+00
6.235754045307E-05	8.226146917273E+00	1.33930000000000E+00
6.790771059360E-05	7.745642156200E+00	1.34230000000000E+00
7.372881629761E-05	7.318066747492E+00	1.34480000000000E+00
7.987951270955E-05	6.928104442245E+00	1.34700000000000E+00
4.635274611399E-05	9.916105331131E+00	1.29630000000000E+00
5.159061358493E-05	9.233161296246E+00	1.30120000000000E+00
5.707488151659E-05	8.623555593398E+00	1.30540000000000E+00
6.286402990654E-05	8.090136406143E+00	1.30880000000000E+00
6.903111797970E-05	7.604410914001E+00	1.31180000000000E+00
7.566707294015E-05	7.171300360729E+00	1.31430000000000E+00
8.287830968910E-05	6.774523518127E+00	1.31650000000000E+00
4.719848757969E-05	1.012387403471E+01	1.34400000000000E+00
5.212561012528E-05	9.428934650150E+00	1.34920000000000E+00
5.715576942092E-05	8.824141683862E+00	1.35340000000000E+00
6.230922895544E-05	8.289772140124E+00	1.35690000000000E+00
6.761567963670E-05	7.810886328114E+00	1.35990000000000E+00
7.310717864044E-05	7.378330822594E+00	1.36250000000000E+00
7.882465712133E-05	6.990133714062E+00	1.36470000000000E+00
