# Supplementary material for: Optimized PCF architectures for THz detection of aquatic pathogens: Enhancing water quality monitoring
Source: PLoS One. 2025 Jan 27;20(1):e0317533. doi: 10.1371/journal.pone.0317533 (PMC11771926; doi:10.1371/journal.pone.0317533)
Supplement: S2 Data — (PDF) [file pone.0317533.s002.pdf]

wave	freq	p	nr	l	aff	real-x pol	real-y pol
9.38E-05	3.20E+12	1.80E-04	1.388	0.00028	0.965	1.35230000000000	
9.38E-05	3.20E+12	1.90E-04	1.388	0.000296	0.965	1.35580000000000	
9.38E-05	3.20E+12	2.00E-04	1.388	0.000311	0.965	1.35880000000000	
9.38E-05	3.20E+12	2.10E-04	1.388	0.000327	0.965	1.36150000000000	
9.38E-05	3.20E+12	2.20E-04	1.388	0.000342	0.965	1.36380000000000	
9.38E-05	3.20E+12	2.30E-04	1.388	0.000358	0.965	1.36590000000000	
9.38E-05	3.20E+12	2.40E-04	1.388	0.000373	0.965	1.36770000000000	
#DIV/0!							
#DIV/0!							
#DIV/0!							
9.38E-05	3.20E+12	1.80E-04	1.365	0.00028	0.965	1.33030000000000	
9.38E-05	3.20E+12	1.90E-04	1.365	0.000296	0.965	1.33370000000000	
9.38E-05	3.20E+12	2.00E-04	1.365	0.000311	0.965	1.33670000000000	
9.38E-05	3.20E+12	2.10E-04	1.365	0.000327	0.965	1.33930000000000	
9.38E-05	3.20E+12	2.20E-04	1.365	0.000342	0.965	1.34160000000000	
9.38E-05	3.20E+12	2.30E-04	1.365	0.000358	0.965	1.34370000000000	
9.38E-05	3.20E+12	2.40E-04	1.365	0.000373	0.965	1.34550000000000	
#DIV/0!							
#DIV/0!							
#DIV/0!							
9.38E-05	3.20E+12	1.80E-04	1.333	0.00028	0.965	1.29990000000000	
9.38E-05	3.20E+12	1.90E-04	1.333	0.000296	0.965	1.30330000000000	
9.38E-05	3.20E+12	2.00E-04	1.333	0.000311	0.965	1.30630000000000	
9.38E-05	3.20E+12	2.10E-04	1.333	0.000327	0.965	1.30880000000000	
9.38E-05	3.20E+12	2.20E-04	1.333	0.000342	0.965	1.31110000000000	
9.38E-05	3.20E+12	2.30E-04	1.333	0.000358	0.965	1.31320000000000	
9.38E-05	3.20E+12	2.40E-04	1.333	0.000373	0.965	1.31500000000000	
wave	freq	p	nr	l	aff	real-x pol	
9.38E-05	3.20E+12	1.80E-04	1.3833	0.00028	0.965	1.34780000000000	
9.38E-05	3.20E+12	1.90E-04	1.3833	0.000296	0.965	1.35130000000000	
9.38E-05	3.20E+12	2.00E-04	1.3833	0.000311	0.965	1.35430000000000	
9.38E-05	3.20E+12	2.10E-04	1.3833	0.000327	0.965	1.35690000000000	
9.38E-05	3.20E+12	2.20E-04	1.3833	0.000342	0.965	1.35930000000000	
9.38E-05	3.20E+12	2.30E-04	1.3833	0.000358	0.965	1.36130000000000	
9.38E-05	3.20E+12	2.40E-04	1.3833	0.000373	0.965	1.36310000000000	

imag-x pol	imag-y	area-x pol	area-y pol	power-x pol	power-y p
2.0589000000000E-18		2.2935000000000E-08		9.4945000000000E-01	
4.4153000000000E-17		2.5104000000000E-08		9.5241000000000E-01	
4.1232000000000E-18		2.7355000000000E-08		9.5483000000000E-01	
7.5780000000000E-17		2.9681000000000E-08		9.5679000000000E-01	
8.0797000000000E-18		3.2074000000000E-08		9.5839000000000E-01	
2.3123000000000E-17		3.4525000000000E-08		9.5966000000000E-01	
3.6189000000000E-17		3.7023000000000E-08		9.6066000000000E-01	
8.7811000000000E-19		2.3104000000000E-08		9.4219000000000E-01	
1.5444000000000E-18		2.5230000000000E-08		9.4526000000000E-01	
3.2032000000000E-18		2.7420000000000E-08		9.4772000000000E-01	
3.5996000000000E-19		2.9664000000000E-08		9.4965000000000E-01	
1.5424000000000E-18		3.1951000000000E-08		9.5116000000000E-01	
3.2195000000000E-18		3.4267000000000E-08		9.5229000000000E-01	
7.8166000000000E-18		3.6597000000000E-08		9.5308000000000E-01	
6.4356000000000E-18		2.3265000000000E-08		9.2953000000000E-01	
1.8225000000000E-17		2.5292000000000E-08		9.3241000000000E-01	
3.1415000000000E-18		2.7351000000000E-08		9.3478000000000E-01	
7.4911000000000E-19		2.9425000000000E-08		9.3676000000000E-01	
1.0445000000000E-18		3.1497000000000E-08		9.3804000000000E-01	
6.3818000000000E-18		3.3547000000000E-08		9.3871000000000E-01	
1.3292000000000E-17		3.5553000000000E-08		9.3890000000000E-01	
imag x pole		area x pole		power-x pol	
6.5022000000000E-18		2.2972000000000E-08		9.4807000000000E-01	
7.9777000000000E-18		2.5134000000000E-08		9.5106000000000E-01	
5.3264000000000E-18		2.7375000000000E-08		9.5348000000000E-01	
2.3291000000000E-17		2.9687000000000E-08		9.5545000000000E-01	
5.5480000000000E-18		3.2061000000000E-08		9.5703000000000E-01	
4.1211000000000E-18		3.4488000000000E-08		9.5829000000000E-01	
1.9871000000000E-17		3.6956000000000E-08		9.5926000000000E-01	

sens-x pol	sens y-pol	EML-x pol	EML-y po	confinement -x pol	confineme
0.9745149744879		0.0010036000000		1.198558312045E-12	
0.9750295618823		0.0048930000000		2.570301867587E-11	
0.9753488666470		0.0048525000000		2.400260154561E-12	
0.9754127947117		0.0048316000000		4.411421093147E-11	
0.9753961871242		0.0048280000000		4.703478359237E-12	
0.9751871147229		0.0048428000000		1.346071390035E-11	
0.9749185347664		0.0048738000000		2.106689336763E-11	
#DIV/0!					
#DIV/0!					
0.9667664060738		0.0055939000000		5.111788039197E-13	
0.9674438779336		0.0056192000000		8.990497144704E-13	
0.9677846936485		0.0056306000000		1.864695704087E-12	
0.9678729560218		0.0056412000000		2.095454126008E-13	
0.9677500000000		0.0056839000000		8.978854439258E-13	
0.9673854655057		0.0057394000000		1.874184509024E-12	
0.9668927536232		0.0058308000000		4.550318569107E-12	
#DIV/0!					
#DIV/0!					
#DIV/0!					
0.9531990845450		0.0010451000000		3.746389758123E-12	
0.9536580449628		0.0068819000000		1.060941533684E-11	
0.9538863507617		0.0010551000000		1.828777957789E-12	
0.9540808985330		0.0070567000000		4.360833537988E-13	
0.9537085805812		0.0072013000000		6.080402918702E-13	
0.9528635622906		0.0073891000000		3.715070880476E-12	
0.9517518631179		0.0010951000000		7.737742038811E-12	
sens-x pol		EML-x pol			
0.9730414238018		0.0050887000000		3.785159967255E-12	
0.9735819566344		0.0050308000000		4.644100561467E-12	
0.9738971306210		0.0049965000000		3.100685314138E-12	
0.9740393433562		0.0049819000000		1.355851262609E-11	
0.9739274619289		0.0049867000000		3.229686490470E-12	
0.9737769463013		0.0011090000000		2.399037670489E-12	
0.9734754295356		0.0050515000000		1.156760999498E-11	

nt-y pol	Total loss-x pol	Total loss-y pol	v para-x pol
	1.003600001199E-03		3.77324735289406E+00
	4.893000025703E-03		3.78501295418936E+00
	4.852500002400E-03		3.79616034574198E+00
	4.831600044114E-03		3.79908229479496E+00
	4.828000004703E-03		3.80494518414118E+00
	4.842800013461E-03		3.80283658537702E+00
	4.873800021067E-03		3.80438829063651E+00
	5.593900000511E-03		3.68935465523279E+00
	5.619200000899E-03		3.70094543218923E+00
	5.630600001865E-03		3.70639227774410E+00
	5.641200000210E-03		3.71041851136171E+00
	5.683900000898E-03		3.71066933971616E+00
	5.739400001874E-03		3.70260769292841E+00
	5.830800004550E-03		3.69796554782135E+00
	1.045100003746E-03		3.56133880409064E+00
	6.881900010609E-03		3.56318890965254E+00
	1.055100001829E-03		3.55827584730962E+00
	7.056700000436E-03		3.55866046188214E+00
	7.201300000608E-03		3.54807902262676E+00
	7.389100003715E-03		3.52842890542068E+00
	1.095100007738E-03		3.51168936348249E+00
	5.088700003785E-03		3.75634168949914E+00
	5.030800004644E-03		3.76690877113358E+00
	4.996500003101E-03		3.77679661549489E+00
	4.981900013559E-03		3.78548907109116E+00
	4.986700003230E-03		3.78285021193030E+00
	1.109000002399E-03		3.78781110929118E+00
	5.051500011568E-03		3.78859715822609E+00

NA- x pol	spot-x pol	nonlinear- x pol
3.29726360629599E-01	1.757723197512E-04	8.065289208633E-05
3.16651081034800E-01	1.853145191216E-04	7.368443594646E-05
3.04602293195916E-01	1.948473111156E-04	6.762105940413E-05
2.93492441877492E-01	2.045292506005E-04	6.232182473636E-05
2.83243304546623E-01	2.141420980586E-04	5.767207333042E-05
2.73785006303174E-01	2.239233860577E-04	5.357781549602E-05
2.65058490386177E-01	2.336226606074E-04	4.996283607487E-05
3.28648920167532E-01	1.773288697207E-04	8.006293628809E-05
3.15938516940515E-01	1.869477292515E-04	7.331645184304E-05
3.04274507695209E-01	1.966726377494E-04	6.746076148797E-05
2.93569282089788E-01	2.064177363077E-04	6.235754045307E-05
2.83744160868845E-01	2.162413827962E-04	5.789409032581E-05
2.74736236878907E-01	2.262649275077E-04	5.398120874311E-05
2.66487762282158E-01	2.362198553600E-04	5.054441839495E-05
3.27632245582992E-01	1.798888146358E-04	7.950887943262E-05
3.15589651494185E-01	1.898417833380E-04	7.313672623755E-05
3.04622499228507E-01	1.999477852206E-04	6.763094877701E-05
2.94655993771166E-01	2.099357612605E-04	6.286402990654E-05
2.85616119802227E-01	2.202050247734E-04	5.872857986475E-05
2.77444201655007E-01	2.307489526935E-04	5.513977643306E-05
2.70090637729598E-01	2.412631685632E-04	5.202863555818E-05
		#DIV/0!
3.29489565351694E-01	1.760787733981E-04	8.052298798537E-05
3.16480985671497E-01	1.856582943061E-04	7.359648603485E-05
3.04501323311547E-01	1.952315895106E-04	6.757165589041E-05
2.93465336204943E-01	2.048113813131E-04	6.230922895544E-05
2.83296115267769E-01	2.146220000712E-04	5.769545803312E-05
2.73920816412061E-01	2.242642402021E-04	5.363529575505E-05
2.65281758532116E-01	2.339961622950E-04	5.005341703648E-05

birefringence

9.635409371564E+00
9.147873752830E+00
8.707350474979E+00
8.301047935747E+00
7.933267147417E+00
7.590862228128E+00
7.279165715324E+00

9.552397354237E+00
9.069290166322E+00
8.627758878982E+00
8.226146917273E+00
7.857217072253E+00
7.513208918468E+00
7.199977085676E+00

9.418912958666E+00
8.933279443660E+00
8.488511703407E+00
8.090136406143E+00
7.717508641557E+00
7.368828873552E+00
7.051001208237E+00

9.618953162631E+00
9.131220663153E+00
8.690473526880E+00
8.289772140124E+00
7.915753207305E+00
7.579459277194E+00
7.267671130771E+00
